# Seasonal and circadian biases in bird tracking with solar GPS-tags

**DOI:** 10.1371/journal.pone.0185344

**Published:** 2017-10-11

**Authors:** Rafa Silva, Isabel Afán, Juan A. Gil, Javier Bustamante

**Affiliations:** 1 Department of Wetland Ecology, Estación Biológica de Doñana (EBD-CSIC), Seville, Spain; 2 GIS and Remote Sensing Lab (LAST-EBD), Estación Biológica de Doñana (EBD-CSIC), Seville, Spain; 3 Fundación para la Conservación del Quebrantahuesos, Zaragoza, Spain; University of Lleida, SPAIN

## Abstract

Global Positioning System (GPS) tags are nowadays widely used in wildlife tracking. This geolocation technique can suffer from fix loss biases due to poor satellite GPS geometry, that result in tracking data gaps leading to wrong research conclusions. In addition, new solar-powered GPS tags deployed on birds can suffer from a new “battery drain bias” currently ignored in movement ecology analyses. We use a GPS tracking dataset of bearded vultures (*Gypaetus barbatus*), tracked for several years with solar GPS tags, to evaluate the causes and triggers of fix and data retrieval loss biases. We compare two models of solar GPS tags using different data retrieval systems (Argos vs GSM-GPRS), and programmed with different duty cycles. Neither of the models was able to accomplish the duty cycle programed initially. Fix and data retrieval loss rates were always greater than expected, and showed non-random gaps in GPS locations. Number of fixes per month of tracking was a bad criterion to identify tags with smaller biases. Fix-loss rates were four times higher due to battery drain than due to poor GPS satellite geometry. Both tag models were biased due to the uneven solar energy available for the recharge of the tag throughout the annual cycle, resulting in greater fix-loss rates in winter compared to summer. In addition, we suggest that the bias found along the diurnal cycle is linked to a complex three-factor interaction of bird flight behavior, topography and fix interval. More fixes were lost when vultures were perching compared to flying, in rugged versus flat topography. But long fix-intervals caused greater loss of fixes in dynamic (flying) versus static situations (perching). To conclude, we emphasize the importance of evaluating fix-loss bias in current tracking projects, and deploying GPS tags that allow remote duty cycle updates so that the most appropriate fix and data retrieval intervals can be selected.

## Introduction

Many wildlife movement studies employ Global Positioning System (GPS) location data [1]. Both basic ecology and conservation studies use location fixes obtained from animal with tags carrying GPS receivers. Among others, there are studies on habitat-selection [2,3], animal behavior [4,5] or human-wildlife conflict [6,7]. However, obtaining a GPS fix, and how reliable this location is, depends on many factors that are not always taken into account in these studies. New GPS-tracking tags tend to give thousands of locations with very high spatial accuracy. They can give the false impression of an unbiased account of the behavior and whereabouts of the animals which carry them. But, if fixes are not obtained at random, more fixes are not necessarily better. This can be specially tricky if animal behavior or habitat use has an influence on the probability of losing a fix.

One potential tracking bias is due to the loss of fixes if the receiver on the tag fails to receive signals from a minimum of three satellites during the limited time the GPS receiver is on, and thus, cannot calculate a fix. This fact, that not all scheduled locations are obtained, has sometimes been called in the literature as “fix-rate bias” [8]. In this work we will use the term “fix-loss rate” (FLR) to describe the rate at which scheduled locations are not obtained, calculated as the inverse of fix success rate, and use the “fix rate bias” to refer to any bias in the fixing rate caused by environmental, technical, or behavioral factors. The existence of bias in the fixes that are routinely loss is an important effect that has to be considered in the analysis of animal movement, and solutions have to be designed to mitigate them [9].

Many studies that have gained an insight into FLR bias have been based on battery operated GPS-tags deployed on terrestrial mammals [8,10–12]. They have concluded that FLR bias is influenced mainly by GPS satellite geometry, fix interval (interval between successive fixes), habitat use, animal behavior, or even GPS-tag position and orientation. It is well known that as the amount of visible sky for the GPS receiver decreases (creating what is known as a poor Geometric Dilution of Precision, or poor-GDOP) the risk of losing the fix increases [13]. Thus, environmental factors that prevent a clear view of the sky, such as rugged topography or canopy closure, have been documented as having an influence on FLR [14,15]. Some studies have shown that a smaller fix intervals yields a smaller FLR [14,16,17]. It has also been demonstrated that inactive animals have higher FLR than active ones [18].

Although these studies have resolved most of the issues in fix rate bias in battery-operated tracking devices used mainly in terrestrial mammals, bias in solar-powered tags frequently used for bird tracking remains virtually unexplored. Solar devices with rechargeable batteries are essential to track birds, which require light long-lasting tags. Birds also move in a three dimensional space and tag retrieval is difficult. Therefore, remote data retrieval systems are also needed, requiring extra battery for data download compared to devices which log the data and are latter recovered. As a result, only devices using solar rechargeable batteries seem able to offer an adequate solution to this problem. To recharge the battery solar tags require solar radiation, which varies extremely over the year, is affected by weather, and can be influenced by device attachment (e.g. be covered by feathers), and bird behavior. Researchers are usually keen to get as much data as possible but intensive schedules can compromise the energy available for tag operation and create data gaps. To date, we have only found two studies mentioning the negative influence of battery drain on FLR [17,19]. In summary, although the trend in tag development has lead towards a new generation of lighter tracking devices with more efficient power solutions, technology has not advanced enough yet so that the risk of losing fixes or losing data retrieval attempts in a non-random way due to an insufficient battery performance can be safely ignored.

Low battery capacity and performance, and poor GDOP are not generally direct causes of poor GPS-tag performance but the resulting effects of a combination of other factors related to the electronics, firmware, and quality of the components. For example, the suboptimal performance of the units can be a consequence of non optimal arrangement of the electronics. Antenna design is often constrained by the size of the units and might not be optimal causing low antenna sensitivity and this increases the time needed for searching satellites and acquiring ephemerides (increasing battery drain). A low antenna sensitivity also reduces the number of satellites received (thus affecting DOP). Battery charge is also determined by the efficiency of the circuitry controlling the solar recharging, and devices from different companies can use different components for their circuitry. All these aspects create differences between tag models that can be evaluated in the lab, but the effect of bird behavior or habitat selection on tag efficiency can only be evaluated with real deployments on birds.

Besides fix interval, the data retrieval interval (interval between successive data retrievals) is another parameter which is usually scheduled in the tag duty cycle. Most tags can store on board the GPS locations and only delete them once successfully transmitted. Depending on the study objectives, data retrieval interval can be critical to achieve success in the tracking program. Species conservation and management programs usually request a quasi-real-time tracking to prevent or quickly identify potential mortality events due to poisoning, accidents with power lines or illegal hunting [20–22], mainly at critical life-stages such as juvenile dispersal or during breeding [23]. This means managers would ask for frequent data retrievals, every 1–2 days in the worst scenario. Argos (www.argos-system.com) Platforms Transmitters Terminals (PTTs) has been the only system available for animal tracking since the 1970s for studies requiring worldwide coverage and in the mid 1990s the system integrated GPS receivers on the PTTs [24] to obtain high-spatial-resolution tracking data. In the last decade, some GPS tags deployed on animals have started to use mobile network data services for data retrieval [25]. GSM/SMS (Global System for Mobile communications/Short Message Service) and the more recent GSM/GPRS (General Packet Radio Service for larger datasets) have emerged as cheaper alternatives to the Argos system. Unfortunately, there are still extensive areas throughout the world (e.g. areas in America, North Asia, Australia, Africa, and practically all oceans) without GSM services [26]. In the same way, data retrieval systems using radio modem technology as VHF/UHF or new protocols like Zigbee or Bluetooth are a solid alternative. They are secure, robust, and cheaper systems, and they require less power [27–29] allowing higher data transfer rates than Argos or GSM. However they are only appropriate for short-range downloads, and hence, inadequate for species with unpredictable movements.

In this paper our aim is to evaluate the existence of FLR bias in solar-powered GPS-tags and determine its causes using data from two tag models deployed on bearded vultures (*Gypaetus barbatus*). Bearded vultures are particularly adequate for this test. They inhabit mountain ranges with a rugged topography that creates difficulties for GPS location. The strongly variable weather conditions, and the seasonal variation in solar radiation makes difficult to predict the performance of solar-powered GPS tags that are frequently used in conservation projects. Bearded vultures require slope winds and thermal updrafts to fly, creating a circadian pattern in flight behavior that could influence the visibility of GPS satellites for the tags. We attempt to find out why GPS tags deployed on bearded vultures lose fixes and if this takes place with a non-random pattern. We evaluate FLR and try to attribute it to "battery drain" (the tag has not enough energy to attempt a fix) or to poor GPS satellite geometry (the tag is not able to contact with enough satellites, poor-GDOP hereafter). We evaluate the influence of other interacting factors such as seasonal variation in solar radiation, fix interval, topography, and bird flight behavior. Our first hypothesis is that FLR in solar tags deployed on bearded vultures is non-random, neither along the year, nor during the daily cycle, and is strongly dependent on the energy availability for the tag. Accordingly, we expect that high cloud coverage and shorter days in winter would produce higher FLR, because solar tags do not fully recharge, and create a seasonal bias in number of fixes acquired. Our second hypothesis is that FLR is also strongly influenced by habitat use and bird behavior. Differences in the intensity of flight behavior along the diurnal cycle expose the tags to different relative satellite-receiver geometries (GDOP), and potentially also to different battery recharge opportunities. In this work we also assess the performance of two different data retrieval systems that have been used (Argos and GSM/GPRS). Related to our first hypothesis, the loss of data retrievals, data retrieval loss rate (hereafter DRLR), should follow the same pattern as FLR throughout the year, as the energy consumption for data transmission is even greater than for fix collection.

## Materials and methods

### Ethics statements

This study was conducted as a part of a long-term conservation and research program leaded by the Fundación para la Conservación del Quebrantahuesos (www.quebrantahuesos.org) in accordance with the competent authorities in the management of this species listed as endangered (EN) in the National Catalogue of Threatened Species of Spain (R.D. 139/2011, BOE n.46, 23^th^ February 2011), as well as in Annexe I of the European Birds Directive (Directive 2009/147/EC) and SPEC-3 category (European threatened species). All the procedures have been specifically carried out with authorization of the Nature Conservation Authority of the Government of Aragon, in compliance with the regional Decree 45/2003 (BOA n.29, 25^th^ February 2003) and following the protocols established by the Strategy for Conservation of the Bearded Vulture in Spain (National Commission for Protection of Nature, 4^th^ June 2000).

### Species and study area

The bearded vulture is a specialized and territorial scavenger feeding mainly on bones and inhabiting rugged mountainous areas, in which takes advantage of slope soaring in order to exploit with low energy costs the large areas that make up their territories. Nests are usually located on remote overhung cliff ledges or in caves. It is an endangered species remaining in a few mountain ranges in Europe, Asia, and Africa [30]. In Europe, the largest breeding natural population is in the Pyrenees (170 breeding pairs), with other smaller populations in Crete and Corsica. There have been successful reintroduction projects in the Alps and Andalusian mountains, where the species was eradicated. The species movements have been studied since the 1980's with the help of conventional radiotracking [31], battery powered Argos PTTs [32], and more recently solar-powered GPS tags [23, 33–35]. Tracking studies have aimed to study the basic movement ecology of the species as well as to solve conservation problems of wild and reintroduced populations (poisoning, lead intoxication, collisions with power lines, and food shortage).

The study area includes two different mountain ranges in the north of the Iberian Peninsula, the natural population in the Pyrenees and reintroduced individuals in the Cantabrian Mountains (S1 Fig). Both regions have a rugged topography, a seasonal climate, and varying weather conditions, with elevations ranging up to approximately 3,300 m in Pyrenees and 2,500 m in Cantabrian Mountains.

### Field procedures

Between 2006 and 2012, 13 bearded vultures (three adults, one immature, and nine nestlings) were tagged with two different models of solar powered GPS-tags (S1 Table). All nestlings were tagged at their nests (reintroduced individuals at the hacking cage) when they were between 85 and 105 days old. Adults and immature birds were trapped with a cannon-net in vulture restaurants. Tags were mounted on backpack-style harnesses using 5 mm silicon cord covered by Teflon ribbon (Bally Ribbon Mills, Bally, Pennsylvania, U.S.A.), following the methodology described by Bögel [36], but not using the weak link. In the Pyrenees (0.85° W 42.51° N), eight individuals (three adults, one immature, and four nestlings) were equipped with GPS-Argos PTT-100 70g tags, hereafter PTT, (Microwave Telemetry Inc., Columbia, Maryland, U.S.A.). From 2010 to 2012 five other bearded vulture nestlings (three in the Pyrenees and two in the Cantabrian Mountains, 6.00° W 42.94° N) were equipped with GPS-GSM/GPRS CTT-1100^1st Gen^ 100g tags, hereafter CTT (Cellular Tracking Technology, LLC., Somerset, Pennsylvania, U.S.A.). Except for the data retrieval system, both, CTTs and PTTs have similar technical characteristics (GPS sensor, solar panels, rechargeable battery, they store-on-board GPS locations until successfully transmitted, etc.). GPS tags were programed with different duty cycles following the recommendation of the manufacturer to obtain the best performance. Once deployed, tags could not be reprogrammed remotely. All PTTs were programed with the same duty cycle (PTT#1), providing a fix every 2 h on a 12 h ON/ 12 h OFF cycle from 7:00 to 19:00 (Coordinated Universal Time, UTC, coincident in the study area with solar time), and a data retrieval every two days. In relation to CTT tags, two were programed in the first year (2010) to provide a fix every 30 s from sunrise to sunset, and data retrieval every day (CTT#1). A light sensor switched the tag off during the night. In the second year (2011) a single CTT was fitted. Due to poor performance of previous schedule, it was programed to provide a fix every 15 min from sunrise to sunset and data retrieval every day if a minimum number of locations, set by the manufacturer, had been recorded, with a maximum data retrieval frequency of once per day (CTT#2). In practice CCT#2 schedule attempted transmission every other day. In the third year (2012), two CTTs were fitted and they were programed to provide a fix every 15 min and data retrieval every day (CTT#3).

All individuals were also equipped with conventional 20 g TW51 VHF radio-tracking transmitters, manufactured by Biotrack (Wareham, Dorset, U.K.; 4-yr battery life expectancy), to allow the birds to be located in case of injury or technical failure of the GPS-tag. Birds were also marked with metal and color darvic rings and patagial/humeral tags. The total weight of all the marks was around 3% of the bird’s body mass, below the generally accepted 5% limit [37].

### Tracking data

To avoid problems with days or months with unequal sampling, we discarded months that were incomplete at the beginning or end of the tracking period for each individual (S2 Table). For individuals tagged as nestlings, we started the tracking period on September 1^st^ of the tagging year, in order to not include data neither during the nesting nor the hacking periods. In the case of individuals tagged as adults or immatures, which were usually trapped in vulture restaurants at any time of the year, we used only data from the month following the deployment date. The end of the tracking period for our analyses was June 30^th^, 2014 for currently active tags and “last data retrieval” date for inactive tags. Only data recorded during the PTT ON cycle were used (07:00–19:00 UTC). Fixes were grouped in 2 h intervals in order to allow comparisons between the two different tag models. We calculated fix and data retrieval loss rates (FLR and DRLR, respectively) per individual as the fraction of scheduled fixes or data retrievals that were lost for each duty cycle. We differentiate in between fixes lost due to battery drain (Battery FLR), and fixes lost due to the tag GPS receiver being unable to find enough satellites to calculate the fix (Geometry FLR), using the information provided by downloaded data. We considered a fix was lost due to battery drain when a scheduled fix was not recorded in the tag data logger or when a timed register without GPS-fix indicated an insufficient battery voltage “low batt”. Regarding fixes lost due to a poor geometry, they were coded as “9999” by CTTs and as “no fix” by PTTs. Additionally, acquired fixes were tagged by both tag models with fix quality: 2D when only three satellites had been used to calculate the fix and no altitude estimate was provided, and 3D when four or more satellites were used to calculate the fix. Only CTTs provided the Horizontal Dilution of Precision (HDOP) for each fix. We calculated the effective time lag between successive fixes and data retrievals to evaluate the performance of the different duty cycles (S3 Table) and compared it to programed schedules. Bird behavior at each fix (classified as perching or flying) was estimated for both tag models on the basis of the instantaneous GPS-speed provided by the tag. Speed threshold between both behaviors was established following a visual inspection of instantaneous GPS-speed histograms of 3D-fixes, taking 1.39 m/s as the limit between perching and flying fixes. Tag battery voltage and bird flying altitude were calculated from the values provided by both tag models for each fix. It is not possible to know where the vulture was or what happened to the GPS-tag when a fix was not recorded, but we assume we can deduce the causes of losing fixes from the quality information provided by recorded fixes.

Data recorded by the tags were automatically uploaded into Movebank (www.movebank.org) within the study named “Bearded Vulture (*Gypaetus barbatus*), Pyrenees and Cantabrian Mountains” through CTT GSM and Argos live feeds in order to follow established recommendations for animal movement data [38].

### Environmental data

We downloaded data on mean monthly potential solar radiation from the digital climate atlas of the Iberian Peninsula [39]. We firstly calculated the 90%-MCP (Minimum Convex Polygon) for each individual using 3D-fixes. Then we extracted the radiation values within each MCP for each tag. Elevation (in meters above WGS 84 ellipsoid) was downloaded from the 30-m spatial resolution ASTER Global Digital Elevation Model (GDEM) v.2 [40] to estimate the vultures' flying altitude above ground level. Elevation data was also used to estimate the terrain roughness, calculated as the standard deviation of the elevation within a 500-m buffer around each 3D-fix.

### GPS tests

GPS theory explains that GPS receivers require more Time To obtain a Fix (TTF) in dynamic than in static conditions [41]. TTF is also shorter when the scheduled fix interval is shorter, because the GPS receiver needs to read ephemeris and almanac data from each GPS satellite being tracked at least once per hour. Depending on the GPS receiver type, collection of ephemeris and almanac data, can take from 30 s to 3 min. Because GPS receivers programed with short fix intervals (below 1 h) can use previously transmitted data, they are able to minimize TTF more than those programed with long fix intervals (above 1 h). A first exploratory analysis of the dataset suggested that there could be an interaction between fix interval and bird behavior (perching or flying) in FLR bias, because PTT tags that have longer fix intervals (2 h) recorded a higher fraction of perching fixes than CTT tags. When the tag is moving and fix interval is long, TTF increases dramatically and there is a higher probability of losing a fix. We designed a field experiment to test for an interaction between fix interval and instantaneous GPS-speed (classified as static or dynamic) in TTF. We used a Garmin GPSMAP^®^ 62s handheld GPS device with the same three fix intervals as programed in the bearded vultures' tags. We measured time since the GPS receiver was switched on until a 3D-fix with an error below 5 m was obtained. 30 TTF measurements were timed with a stopwatch for each state (static and dynamic) and fix interval (30 s, 15 min and 2 h) (n = 180) by performing alternative stationary and dynamic tests. Stationary tests were always conducted at the same point (6.56° W, 37.89° N) in order to avoid the bias produced by topography. Dynamic tests were carried out driving the GPS on a car on a fixed 10-km route at a speed of 50 (km/ h). Maximum GPS timeout was established as 180 s. After this time, if no fix was achieved, the GPS was turned off and the fix was taken as lost.

### Statistical analysis

To estimate the potential seasonal and circadian biases in fixes and data retrievals attempts we fitted Generalized Linear Mixed Models (GLMMs). We used Battery FLR (fixes lost due to battery / fixes scheduled), Geometry FLR (fixes lost due to poor GDOP / fixes scheduled) and DRLR (data retrieval lost / data retrievals scheduled) as response variables in the models with “binomial” errors and “logit” links. To test for the existence of a seasonal bias in FLR and DRLR due to battery drain, we fitted a GLMM using as response variables Battery FLR and DRLR, and as predictor variables the tag model and month, including the individual as a random factor. To test for the existence of a circadian bias in FLR due to poor-GDOP, we fitted a GLMM using as response variable Geometry FLR, and as predictor variables tag model and solar time (Hour UTC), including the individual as a random factor. Statistical significance of predictors was made with an ANOVA test, using the Chi-squared test to select among alternative models.

To determine the relationship between solar radiation and battery voltage, using mean monthly values for each individual tag, we used linear regression analysis. The influence of bird flight behavior (based on the instantaneous GPS-speed classification as “perching” or “flying”) on Geometry FLR was examined with linear regression analysis. We related HDOP with terrain roughness and vultures' flying altitude for fixes classified as "perching" and "flying" respectively. Previously, we tested with a Mann-Whitney’s U test, if there were any differences in mean instantaneous GPS-speed values between both tag models. To analyze our field GPS experiment, we used a two-way ANOVA to test for differences in TTF in relation to fix interval and state (static and dynamic) and also its interaction. To avoid having an unbalanced dataset, we conservatively used TTF = 180 s for the fixes that were not acquired using the maximum GPS timeout.

All data analyses were performed with R v. 3.2.4 [42] accessing to movebank data store by R package “move” [43].

## Results

The 13 bearded vultures tracked in a 7 year period from 2007 to 2014 provided a total of 83,231 GPS fixes (48.1% were recorded by CTTs and 51.9% by PTTs tags). CTT tags provided an average of 256.3 fixes per month of tracking, while PTT tags provided an average 126.6 fixes per month of tracking (S1 and S2 Tables).

### Tracking efficiency

**Fig 1:**
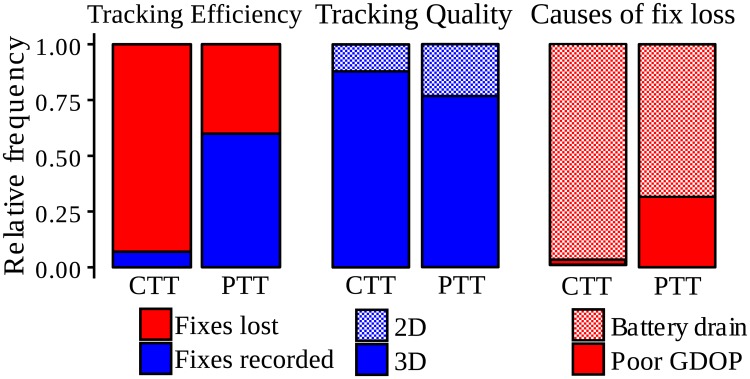
Tracking efficiency, tracking quality and causes of fix loss of GPS solar tags. Tag models: Celltracktech GSM-GPRS (CTT) and Microwave Argos (PTT). Tracking Efficiency: mean rate of scheduled fixes that were acquired (blue) and lost (red). Tracking Quality: Based on acquired fixes (blue), the percentage of real fixes that are 3D fixes (solid) versus 2D fixes (hatched). Based on lost fixes (red), the percentage that were lost due to battery drain (no record, or tagged as "low batt") (solid), and due to poor-GDOP (record with no location, or tagged as "timeout") (hatched).

CCTs were programed to provide, depending on duty cycle, from 2,880 (CTT#1) to 48 (CTT#3) fixes per day, while PTTs were all programed to provide 7 (PTT#1) fixes per day. Neither tag model accomplished this task. Regarding days-with-fixes, CTTs only collected fixes in 31.88 ± 33.30% (mean ± SD) of the days of tracking, providing a FLR of 0.93 ± 0.07, while PTTs collected fixes 85.54 ± 10.67% of the days, providing a FLR of 0.40 ± 0.12 (Fig 1 and Table 1). Gaps between fixes were usually longer and more frequent in CTTs than in PTTs. The maximum period without fixes ranged from 12 to 307 days for CTTs, and from 7 to 11 days in PTTs. The standard deviation of the time lag between successive fixes was 83.65 h for CTTs and 7.68 h for PTTs. With regard to the location accuracy of GPS fixes (estimated as the percentage of 3D fixes from total fixes), CTTs provided more accurate locations (% 3D fixes = 88% ± 9%) than PTTs (% 3D fixes = 77% ± 6%).

**Table 1:** Fix loss rate (FLR), data retrieval loss rate (DRLR) and performance of individual GPS solar tags.

Individual	Duty cycle	Days with fix (%)	FLR	%3D	Time-lag		DRLR	Time-lag (days)	
					max (days)	SD (hours)		max	SD
Carrodilla	PTT#1	61	0.66	78	9	13.86	0.46	22	2
Asterix	PTT#1	88	0.38	75	8	5.35	0.12	14	1
Goriz	PTT#1	91	0.39	75	9	6.95	0.18	14	1
Rover	PTT#1	83	0.33	90	8	7.59	0.20	10	1
Eva	PTT#1	87	0.45	73	11	8.31	0.21	12	1
Ixeia	PTT#1	87	0.44	69	11	8.36	0.19	14	1
Sevil	PTT#1	96	0.3	76	7	5.59	0.11	10	1
Maria	PTT#1	91	0.26	78	7	5.41	0.11	10	1
PTT mean		86	0.4	77	9	7.68	0.20	13	1
PTT SD		11	0.12	6	2	2.79	0.11	4	0
Deva	CTT#1	16	0.99	89	243	65.79	0.85	246	25
Coto	CTT#1	7	0.99	96	230	85.19	0.93	230	40
Luisa	CTT#2	90	0.88	93	12	8.49	0.93	59	8
Cotiella	CTT#3	24	0.82	90	182	62.15	0.80	183	18
Atilano	CTT#3	22	0.96	73	307	196.62	0.93	310	49
CTT mean		32	0.93	88	195	83.65	0.89	206	28
CTT SD		33	0.07	9	111	69.27	0.06	94	16

Duty cycle: PTT#1 = one fix every 2 h on a 12 h ON/ 12 h OFF cycle from 7:00 to 19:00 UTC, Data retrieval every 2 days, CTT#1 = one fix every 30 s from sunrise to sunset. Data retrieval every day, CTT#2 = one fix every 15 min from sunrise to sunset. Data retrieval once a certain number of fixes are stored, CTT#3 = one fix every 15 min from sunrise to sunset. Data retrieval every day. Days with a fix (% of days obtaining at least one GPS fix), FLR = Fix Loss Rate (fraction of scheduled fixes that are lost), % 3D (% of fixes that are acquired with three or more satellites and have altitude information). DRLR = Data Retrieval Loss Rate (fraction of scheduled data retrievals that are unsuccessful). Fix Time-lag = time lag between consecutive fixes. Retrieval Time-lag = time lag between consecutive data retrievals.

A better performance on data retrieval was found in PTTs (DRLR = 0.20 ± 0.11) than in CTTs (DRLR = 0.89 ± 0.06). The time lag between successive data retrievals was closer in PTTs to the programed schedule (scheduled every two days, mean retrieval time-lag = 2.44 ± 1.32 days) than in CTTs (scheduled every day, mean retrieval time-lag = 10.54 ± 27.93 days). Retrieval time-lags ranged between 10–22 days for PTTs and 59–310 days for CTTs (Table 1).

### Seasonal and circadian biases in fix and data retrieval loss rates

Fix-loss rate (FLR) was in general more noticeable due to battery drain (90% of the fixes lost by CTTs and 68% of those lost by PTTs) than due to poor-GDOP (10% for CTTs and 32% for PTTs) (Fig 1).

#### Seasonal bias

**Fig 2:**
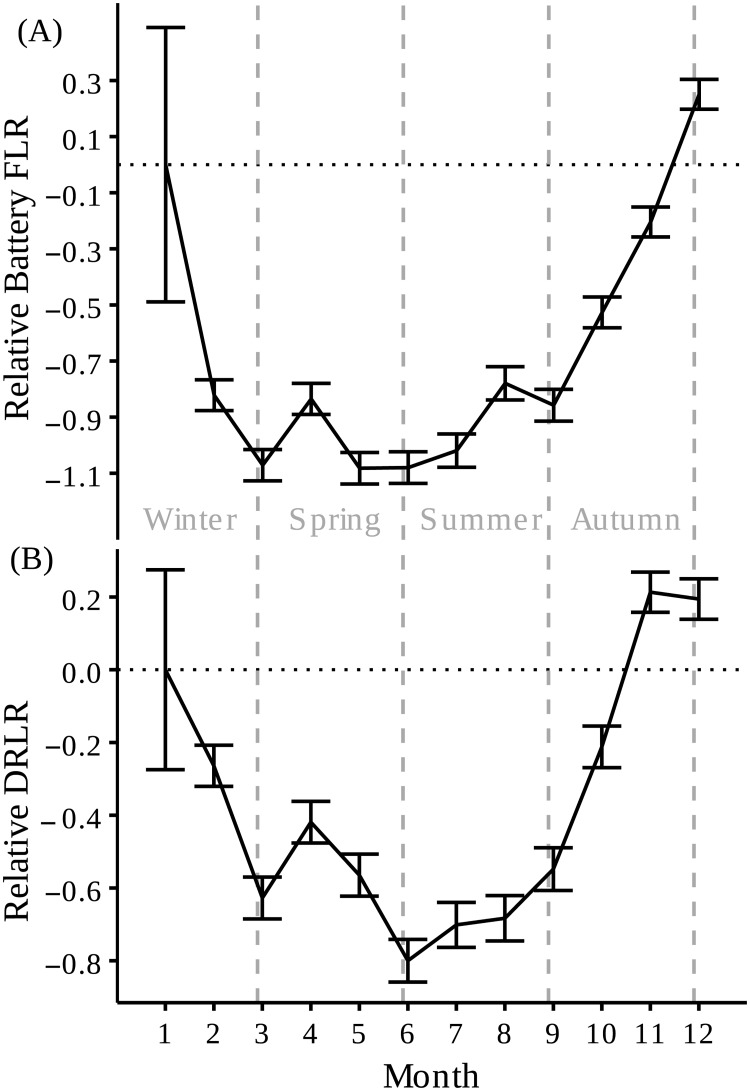
Seasonal tracking bias due to battery drain predicted by the GLMM. Mean monthly regression coefficients (solid line), with standard error bars. (A) Relative Battery FLR = fix-loss rate due to battery drain, and (B) Relative DRLR = data retrieval-loss rate. January was fixed as intercept.

Seasonal analyses with GLMMs showed a significant effect of the month and the tag model in Battery FLR and in DRLR (Table 2), with more fixes and data retrievals losses in autumn and winter than in spring and summer (Fig 2 and S2 Fig). Best GLMMs estimated that Battery FLR was 4 times higher (4.29 ± 0.62, mean ± SE), and that DRLR was almost 4 times higher (3.73 ± 0.34) in CTTs than PTTs, due to the uneven recharge of the battery along the year.

**Table 2:** GLMMs fitted to fix loss rate due to battery drain (Battery FLR) and to data retrieval loss rate (DRLR) to evaluate seasonal bias.

Model	Deviance	χ^2^	Df	p-value
Battery FLR ~ + (1|Individual) *Null model*	92929.92			
**Battery FLR ~ Month + (1|Individual)**	**72096.11**	**20833.81**	**11**	**<0.001**
Model	Deviance	χ^2^	Df	p-value
DRLR ~ + (1|Individual) *Null model*	3366.57			
DRLR ~ Month + (1|Individual)	3153.18	213.39	11	< 0.001
**DRLR ~ Month + Tag model + (1|Individual)**	**3123.4**	**29.77**	**1**	**< 0.001**

Best models (in bold) were selected by Chi-squared test.

#### Battery voltage

**Fig 3:**
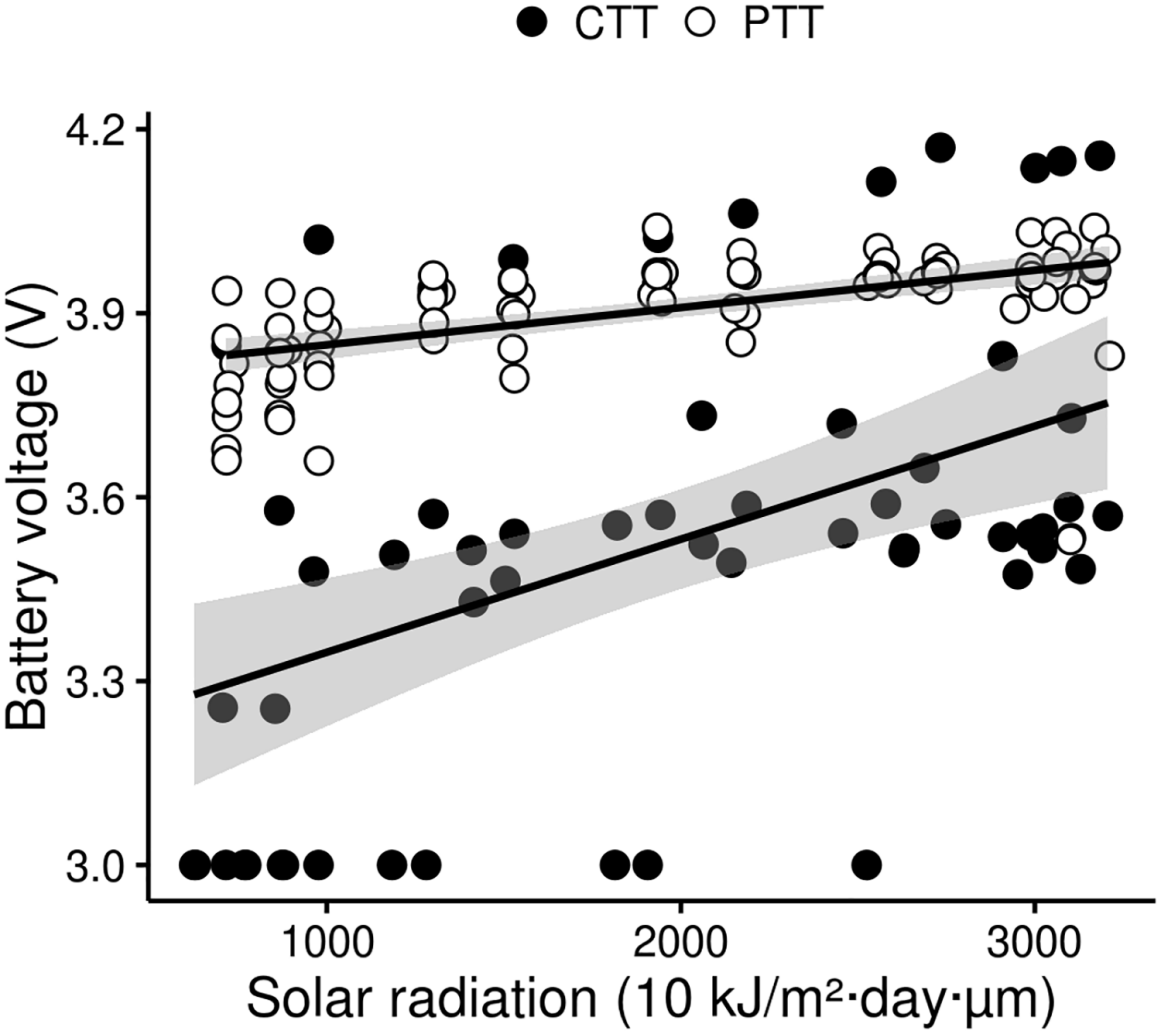
Effect of solar radiation on the tag battery performance. Least squares linear regression lines and 95% confidence intervals (represented as a gray shadow) for mean monthly battery voltage (Battery Voltage) predicted from the mean monthly potential solar radiation (Solar Radiation) for each tag per month of tracking. Celltracktech GSM-GPRS tags (CTTs, n = 5) are represented as black dots (r = 0.45, p < 0.05, n = 60) and Microwave Argos tags (PTTs, n = 8) are represented as white dots (r = 0.68, p < 0.05, n = 96).

Although the incident mean solar radiation was equal for all tags (S3A Fig), battery voltage was on average lower in CTTs than in PTTs (S3B Fig). The results of regression analyses showed a positive significant correlation between mean monthly solar radiation and mean monthly battery voltage (r = 0.41, p < 0.05, n = 156), both in PTTs (r = 0.68, p < 0.05, n = 96) and in CTTs (r = 0.45, p < 0.05, n = 60, Fig 3).

#### Circadian bias

**Fig 4:**
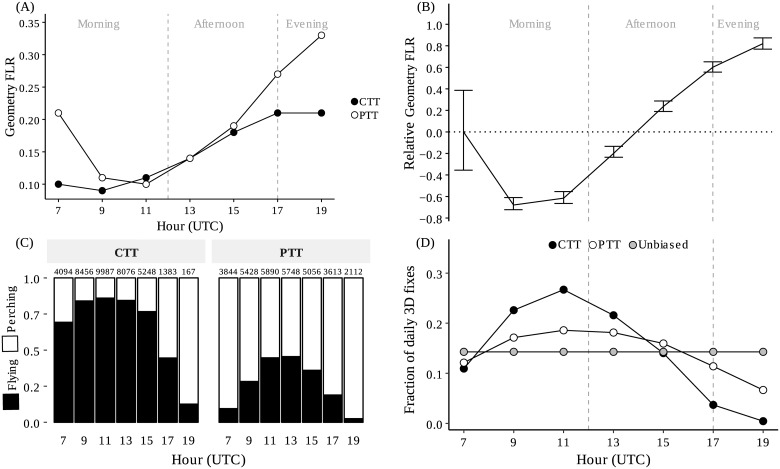
Circadian tracking bias due to poor GDOP and circadian pattern in flight behavior. (A) Fraction of fixes lost due to poor-GDOP (Geometry FLR) for each model in 2-h intervals. (B) GLMM regression coefficient for Geometry FLR for each 2-h interval (solid line), with standard error bars. Intercept was fixed at 7:00 am. (C) The stacked bars represent the mean % of 3D-fixes classified as “perching” or “flying” using the instantaneous GPS speed in 2-h intervals throughout the day. The total number of 3-D fixes is shown on top of each bar. Data provided by Celltracktech GSM-GPRS tags (CTTs, n = 5) Microwave Argos tags (PTTs, n = 8). (D) Mean fraction of total daily 3D fixes acquired at each 2-h interval for CTTs and PTTs tags. Grey dots represent a hypothetical unbiased and 100%-efficient tag model that would acquire 1/7th of the 3D-fixes on each seven 2-h intervals.

Circadian analyses with GLMMs showed a significant effect of solar time in Geometry FLR. GLMMs highlighted a bias throughout the diurnal cycle. More fixes were lost due to poor-GDOP in the afternoon than in the morning and the greatest number were lost close to dusk. The pattern was similar in both CTTs and PTTs (Fig 4A and 4B). Only the time of day showed a statistical significant effect, with no significant difference between tag models (Table 3).

**Table 3:** GLMMs fitted to fix loss rate due to poor GDOP (Geometry FLR) to evaluate circadian bias.

Model	Deviance	χ^2^	Df	p-value
Geometry FLR ~ + (1|Individual) Null model	37218.63			
**Geometry FLR ~ Hour + (1|Individual)**	**35894.71**	**1323.91**	**6**	**< 0.001**
Geometry FLR ~ Hour + Tag model + (1|Individual)	35892.79	1.92	1	0.16

Best models (in bold) were selected by Chi-squared test.

#### Terrain roughness

A Spearman's correlation test showed a positive significant correlation between terrain roughness and HDOP (r = 0.12, p < 0.001, n = 7,398) for the fraction of 3D fixes classified as "perching", and a significant negative correlation between flying altitude and HDOP (r = −0.15, p < 0.001, n = 28,451) for the fraction of 3D fixes classified as “flying”.

#### Flight behavior

Instantaneous GPS-speed of 3D fixes allowed us to differentiate between “perching” and “flying” behavior. This allowed us to estimate the effect of bird behavior in FLR. The mean speed of fixes classified as “perching” was 0.01 m/s, (n = 29,391) while for those classified as “flying” 21.1 m/s, (n = 39,711), with no significant differences between tag models (U = 8976871, p < 0.4096). The comparison of fixes classified as “flying” versus “perching” indicated a different flight activity time budget in bearded vultures according to tag model. Using all tracking data, CTTs collected a 80% of 3D- fixes in flight, while PTTs collected only 20%. The circadian pattern in flight behavior, with higher flight activity around noon, was apparent for both tag models (Fig 4C), but suggested a different proportion of flying vs. perching behavior. Throughout the day, both CTTs and PTTs recorded a higher percentage of 3D fixes at noon than either during the morning or evening (Fig 4D). When we compared % 3D fixes for each 2-h interval, PTTs were closer to a hypothetical unbiased and 100%-efficient tag model (FLR = 0), i.e., which must acquire the same amount of 3D fixes at each 2-h interval, conforming with the regular fix interval programed.

#### Interaction effect of GPS speed and fix interval

**Fig 5:**
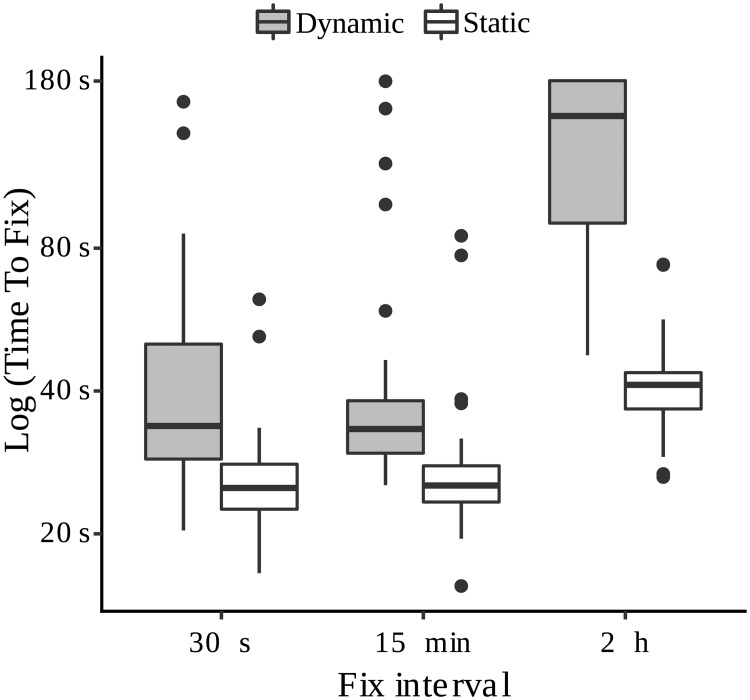
Interaction effect on time to fix (TTF) of fix interval and the static or dynamic state of the GPS tag. TTF = time to fix in seconds (log scale) to acquire a 3D-fix (GPS error < 5 m) for each fix interval programed (30 s, 15 min and 2 h) and per state (static or dynamic, n = 180 tests), using a handheld Garmin GPSMAP^®^ 62s.

The two-way ANOVA performed on the sample of our field experiment showed a significant interaction between instantaneous GPS-speed (classified as static or dynamic state) and fix interval (30 s, 15 min and 2 h) (Table 4). Field GPS tests demonstrated that the effect of the longest fix interval on the fix-rate bias is higher in a dynamic test (when GPS is moving) than in stationary test (Fig 5). Our experiment indicates that there were no significant differences in time to fix (TTF) between 30 s and 15 min fix intervals, that needed on average 26.94 ± 9.31 s and 29.03 ± 14.94 s, respectively, in a static and 46.89 ± 32.83 s, and 47.14 ± 39.01 s in a dynamic state. In both cases (30 s and 15 min) all the fixes were achieved (FLR = 0). In contrast, the 2 h fix interval needed 42.28 ± 10.84 s in static and 136.71 ± 48.85 s in dynamic state, with a FLR of 0.22.

**Table 4:** Results of the two-way ANOVA to test the interaction between fix interval and static/dynamic state in time to fix (TTF).

Response variable: Time to fix (TTF)	Df	Sum Sq	Mean Sq	F Value	p-value
State (Static / Dynamic)	1	87776.83	87776.83	97.28	< 0.001
Fix interval	2	108163.29	54081.64	59.93	< 0.001
State * Fix interval	**2**	**56879.78**	**28439.89**	**31.52**	**< 0.001**
Residuals	174	157006.27	902.33		

Best models (in bold) were selected by F test.

## Discussion

**Fig 6:**
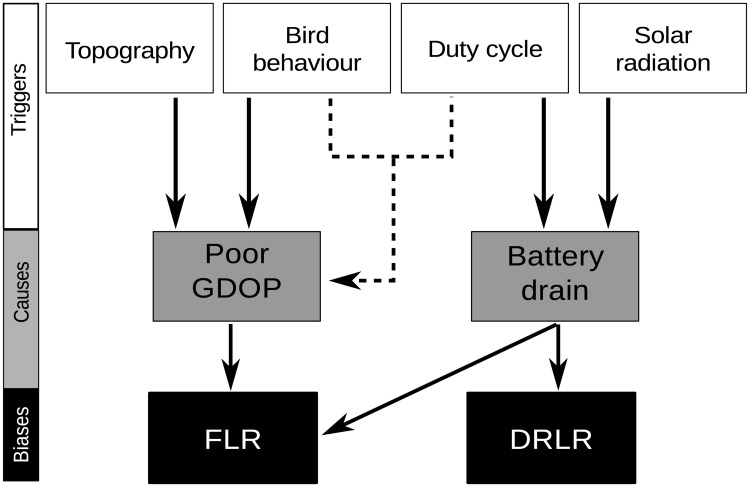
Relationships between triggers and causes of bias in fix-loss rate (FLR) and data retrieval-loss rate (DRLR) considered in this study. Black arrows show how triggers influence the proximate causes of FLR and DRLR. Interactions between triggers are indicated by dashed arrows.

Our results demonstrate that none of the tag models was able to accomplish the duty cycle programed initially. Fix success rate and data retrieval success rate were always below expectations, showing non-random gaps that indicate a bias in tracking data. If number of fixes had been the aim of the tracking program, CTT tags provided more fixes per month of tracking than PTT tags. But the CTTs tested in this study, especially the devices with intense schedules, showed higher values of FLR and DRLR than PTTs suggesting greater biases in tracking information. Battery drain was the main cause of fix loss being responsible for 80% of the losses while 20% were due to a poor-GDOP (Fig 1). Supporting our first hypothesis, FLR bias due to battery drain was closely related to seasonal variation in solar radiation intensity and fix schedule intensity. During autumn and winter, with more cloudy skies and shorter days in the study area, FLR increases due to the battery voltage values falling below the critical disconnection threshold. Consequently, the tag automatically hibernates, turning off the GPS receiver until optimal battery conditions are obtained (Figs 2 and 3). A similar pattern was revealed in relation to DRLR. GLMMs showed the existence of a significant effect of the month and also of the tag model in FLR due to battery drain and DRLR. Thus, gaps (in fixes and successful data retrievals) increase significantly in tags with demanding duty cycles. This was noticeable in tags with duty cycles with short fix intervals (30 s and 15 min) and short data retrieval intervals (1 day) as the Celltracktech GSM-GPRS tags (CTTs) compared to the milder duty cycles (2 h and 2 days, respectively) programed in the Microwave Argos tags (PTTs). Supporting our second hypothesis, we found a circadian bias in FLR due to poor GPS-satellite visibility. Solar radiation also shows a circadian variation and could influence battery charge, but more fixes were lost in the evening (when the tag should be fully charged) than in the morning (after a night period of no battery recharge). And there was no difference between tag models (suggesting no effect of duty cycle demand). This effect in FLR due to poor-GDOP pattern is likely linked to a complex three-factor interaction of bird behavior, topography, and fix interval. In this sense, bird flight behavior and topography demonstrated opposite effects on FLR due to poor-GDOP. On the one hand, our results suggest that more fixes were lost close to dawn and dusk than at noon (Fig 4A and 4B), likely due to the fact the vultures usually spend more time perched at the start and end of the day, when flight condition for a soaring raptor are poor (Fig 4C), increasing the probability that topography could hinder a good GPS signal. In addition, regression analyses also highlighted a significant correlations between HDOP and terrain roughness, and also between HDOP and flying altitude, suggesting that the risk of losing fixes due to poor-GDOP is higher when the vultures remain perched in areas of rough topography and decreases, conversely, as they fly higher over the terrain. However, our GPS test suggested that vulture flight behavior could have an opposite effect on FLR, increasing when the birds move (dynamic versus static state). In addition, TTF increased significantly more with the longest fix interval in a dynamic rather than in a static state (Fig 5). As a consequence, when bearded vultures are flying under optimum GPS-satellite visibility, PTTs tags that were scheduled with 2 h fix intervals could also be losing more fixes than CTTs (that have 30 s or 15 min fix intervals), because of the interaction between tag speed and fix interval. The opposite situation could be happening when vultures are perched. PTTs would be losing less fixes than CTTs because in this situation the longer fix interval is not so relevant. Fig 6 tries to summarize the interaction between different factors on FLR and DRLR. Topography and bird behavior influence GDOP causing fixes to be lost in a non-random way. Seasonality in solar radiation and weather influences battery recharge and causes fixes to be lost and unsuccessful data retrievals to be more frequent in winter. An intensive duty cycle influences battery discharge causing fixes to be lost, but also interacts with bird behavior because long fix intervals require longer times to obtain a fix if the vulture is flying versus perching. FLR and DRLR show a seasonal bias due to the uneven solar energy available for the recharge of the tag. This explains why we are getting more fixes in summer than in winter and this would have to be taken into account when estimating seasonal movement rates and in any movement variable that is influenced by FLR. In the same way circadian biases in FLR preclude a good estimate of flight activity budget throughout the diurnal cycle. Short and long scheduled fix intervals showed bias in the same direction (underestimating time perching) but probably with a different intensity. CTTs underestimated time perching more than PTTs. Short fix intervals dramatically affect the battery level and, therefore, were only able to perform satisfactorily when the birds were flying high under optimal conditions of solar radiation, avoiding, incidentally, the effect of topography. Long fix intervals also suffered the effect of topography, underestimating the time perched, but with less intensity due to a better battery management. In contrast, long fix intervals could be underestimating the time flying due to the interaction of TTF and flight speed.

The effect of topography on FLR had been previously documented with GPS tags tests placed at varying rugged areas in Arizona [14]. Similarly, behavioral influences on FLR had also been observed using activity sensors on elk (*Cervus canadensis*) [44] and moose (*Alces alces*) [45], to conclude that inactive animals had higher FLR. The same pattern has been observed in lynx (*Lynx lynx*) and wolverines (*Gulo gulo*) during periods of low activity in northern Sweden [46]. Regarding the fix interval effect, several works have demonstrated an inverse relation between fix interval and FLR, accounting for 53% of the variation in FLR as described in a 35-articles review by Cain III et al. [14]. As a direct consequence of this, distance traveled and territory sizes of eastern timber wolves (*Canis lupus*) in Ontario were alarmingly underestimated as fix interval was increased [17]. Potential systematic bias in GPS tracking data has been documented in relation to topography, vegetation, animal behavior, and fix interval on FLR, resulting in the underrepresentation in the use of certain areas [14], certain animal behaviors on activity time budgets [44,45,46], or even movement metrics like cumulative daily distance traveled [17].

In bearded vulture movement ecology this circadian and seasonal biases in fix loss rate should not be disregarded. Our results indicate that time budget estimates of flight behavior can be from slightly to severely biased depending on tag model and duty cycle used. Seasonal biases will affect any movement variable like home range or distance moved that are influenced by fix-rate. Margalida et al. [35] concluded that there were no differences between the breeding and non breeding period in home range or distance moved in Pyrenean bearded vultures while Kruger et al. [34] concluded that adult bearded vulture home ranges did not vary between the breeding and non breeding period in South Africa. As all their data are based on solar-powered PTT tags that we know show differential monthly biases depending on solar irradiation, and those variables are influenced by fix rate, we could expect that home range and distance moved could be underestimated in winter. Winter solstice insolation is 59% of that of summer at 29° South (South Africa) and 42% of summer at 40° North (Pyrenees). Having months with high and low fix-loss rates in both breeding and non-breeding periods could reduce the possibility of finding statistical differences. Comparisons of movement variables of individuals tagged with different solar and battery powered tag models, duty cycles, and in different mountain ranges should also be done with caution. Margalida et al. [33] used only one fix per day in his analysis of distance moved by Bearded vultures. This can be a wise way to solve the problem of FLR bias, but still the solar devices we tested had days without fixes recorded (14.5% of days without fixes in Argos PTTs) and some movement variables could be biased. Considering that solar-powered tags are currently the only alternative for long-time monitoring of bearded vultures, attention should be paid to their performance and biases. Back-pack attachment methods currently used seem to preclude an efficient recharge of the tags due to frequent feather coverage of the solar panels when birds are perching and sometimes even in flight (S4 Fig).

Bias correction methods as sample weighting or iterative simulations have been proved useful in reducing erroneous conclusions in habitat selection studies [9]. However, the best correction factors may also vary between tag models, species, populations, and individuals according to range use and movement patterns. These bias models should not be extrapolated from other study areas, but should be developed ad hoc using the same tags, environmental conditions, and fix intervals that are going to be used for the correction.

We note that a new “battery drain bias” in fix and data retrieval loss rates should be considered in free ranging animal studies where solar-powered tags (equipped with remote data retrieval systems) are the only possible way.

Managers and researchers usually do not have identical requirements in species tracking and, therefore, the specific requirements of the tags and duty cycles can vary noticeably according to monitoring objectives. We advise that a thorough evaluation of the study objectives and choices of tracking system could save significant time and money. The main two aspects in the choice of GPS tracking system are usually the costs and the weight of the tags. Power consumption, data transfer speed or the ability to reprogram the duty cycle remotely are usually ignored. However, the life of a tag lasts as long as the battery recharges and this, in turn, is mainly conditioned by the amount of data stored that need to be downloaded. In this respect, we suspect that the bad performance of CTT tags in high demanding duty cycles is due to the amount energy required to transmit the large volume of data stored when they were collecting fixes. In this case, fix and transmission gaps reached values above 300 days, precluding an adequate monitoring of the bearded vultures reintroduced in the Cantabrian mountains (S3 Table). If there is no option to reschedule the duty cycle of the tag, the case is destined for failure. For that reason, in addition to a good knowledge of the energy consumption of the tag, we emphasize the importance of using tags that allow remote duty cycle updates to adjust, at any time the optimum fix and data retrieval intervals for the project goals, thus also removing some potential sources of bias.

## Supporting information

S1 Fig Location of the two study areas. Cantabrian Mountains (B) and The Pyrenees (C). The GPS fixes (83,231) used in this study are shown as small dots. Two bearded vultures were tracked in the Cantabrian Mountain (B) using CTT tags and 11 were tracked in the Pyrenees (C) (three CTTs and eight PTTs).
(PDF)

S2 Fig Seasonal distribution of loss rates due to battery drain. (A) Mean monthly fix-loss rate due to battery drain (Battery FLR). (B) Mean monthly data retrieval-loss rate (DRLR). In both, (A) and (B) Celltracktech GSM-GPRS tags (CTTs, n = 5) are represented as black dots and Microwave Argos tags (PTTs, n = 8) are represented as white dots.
(PDF)

S3 Fig Solar radiation and tag battery voltage. (A) Mean Monthly Potential Solar Radiation (Solar radiation). (B) Mean Monthly Battery Voltage (Battery voltage). In both, (A) and (B) Celltracktech GSM-GPRS tags (CTTs, n = 5) are represented as black dots and Microwave Argos tags (PTTs, n = 8) are represented as white dots.
(PDF)

S4 Fig Photos of bearded vultures with solar-powered GPS-tags perched and in flight. On the left individuals perching, on the right individuals flying. Frequently feathers are observed totally or partially covering the solar panels or even the tracking device completely. (C) Javier Gil Vaquero/ F.C.Q.
(PDF)

S1 Table Summary of individual bearded vultures tracked in this study. Age code at deployment date: nestling (1), immature (3) or adult (4). Sex as F (Female) or M (Male).
(PDF)

S2 Table Duration of the tracking period for each individual included in the study. Number of GPS fixes recorded refers to the total amount of GPS data acquired by each individual for the entire tracking period.
(PDF)

S3 Table Data retrieval and fix time lags obtained per individual. (PDF)
