# Supplementary figures and images for: Seasonal and circadian biases in bird tracking with solar GPS-tags
Source: PLoS One. 2017 Oct 11;12(10):e0185344. doi: 10.1371/journal.pone.0185344 (PMC5636103; doi:10.1371/journal.pone.0185344)

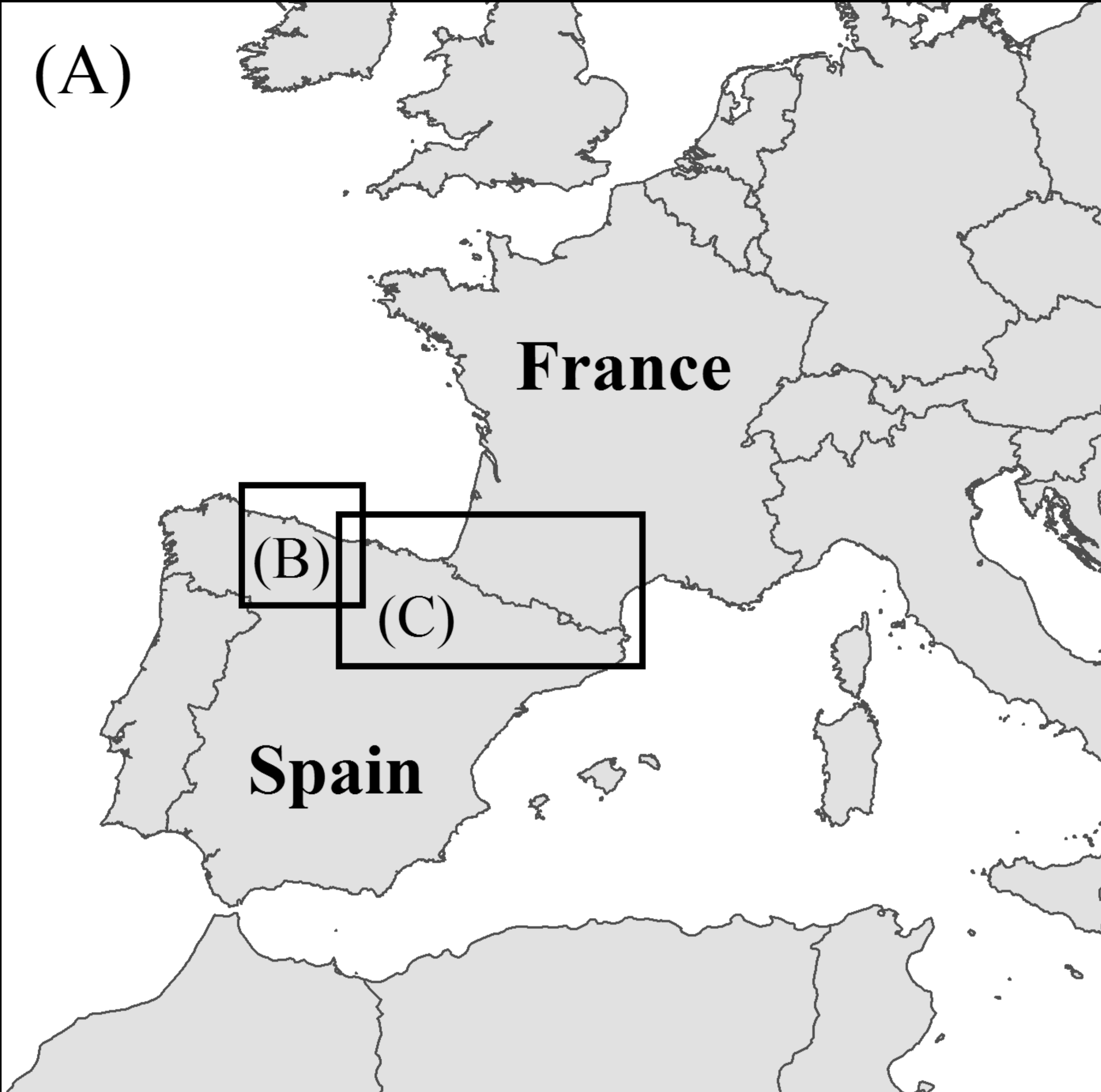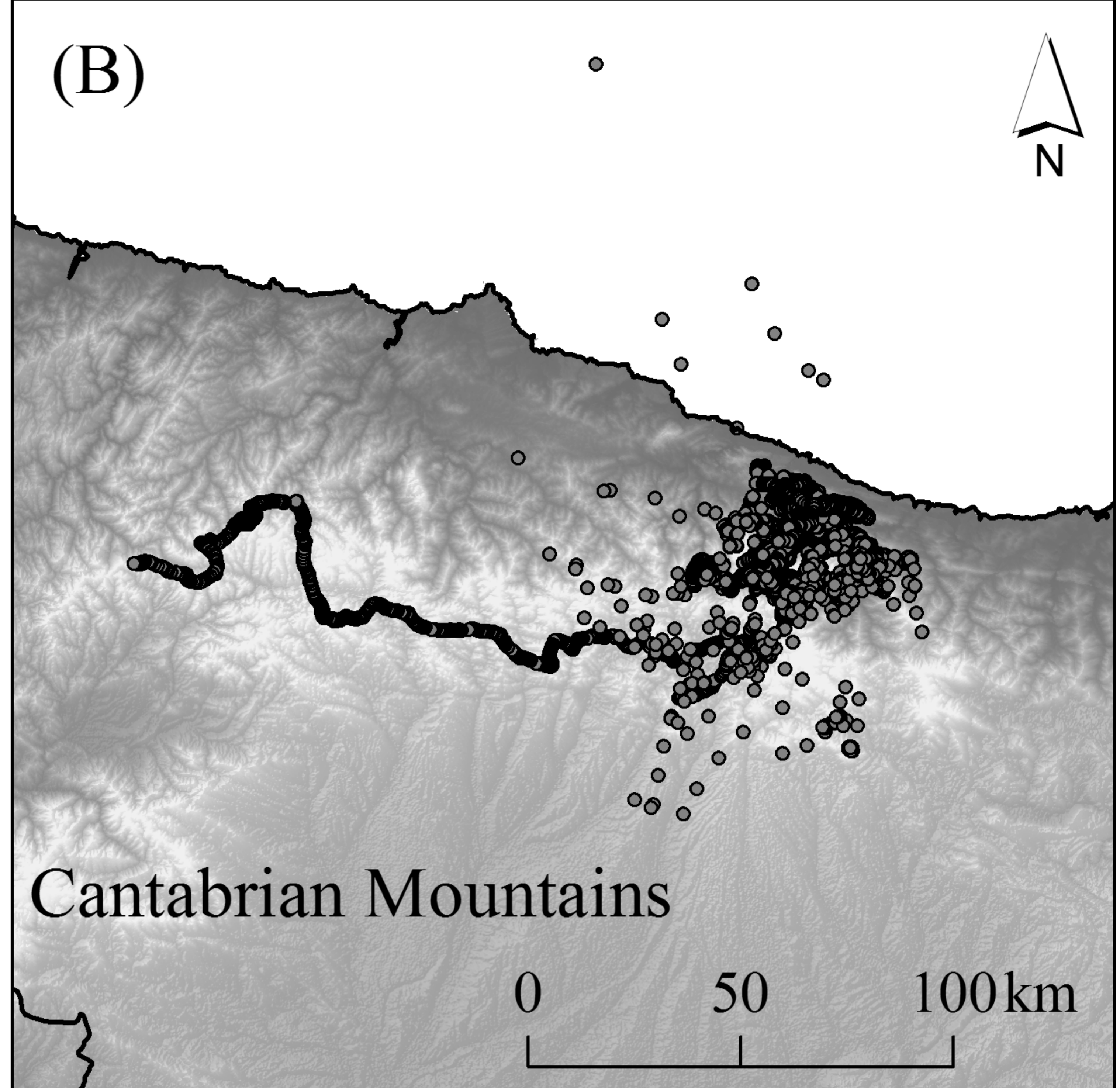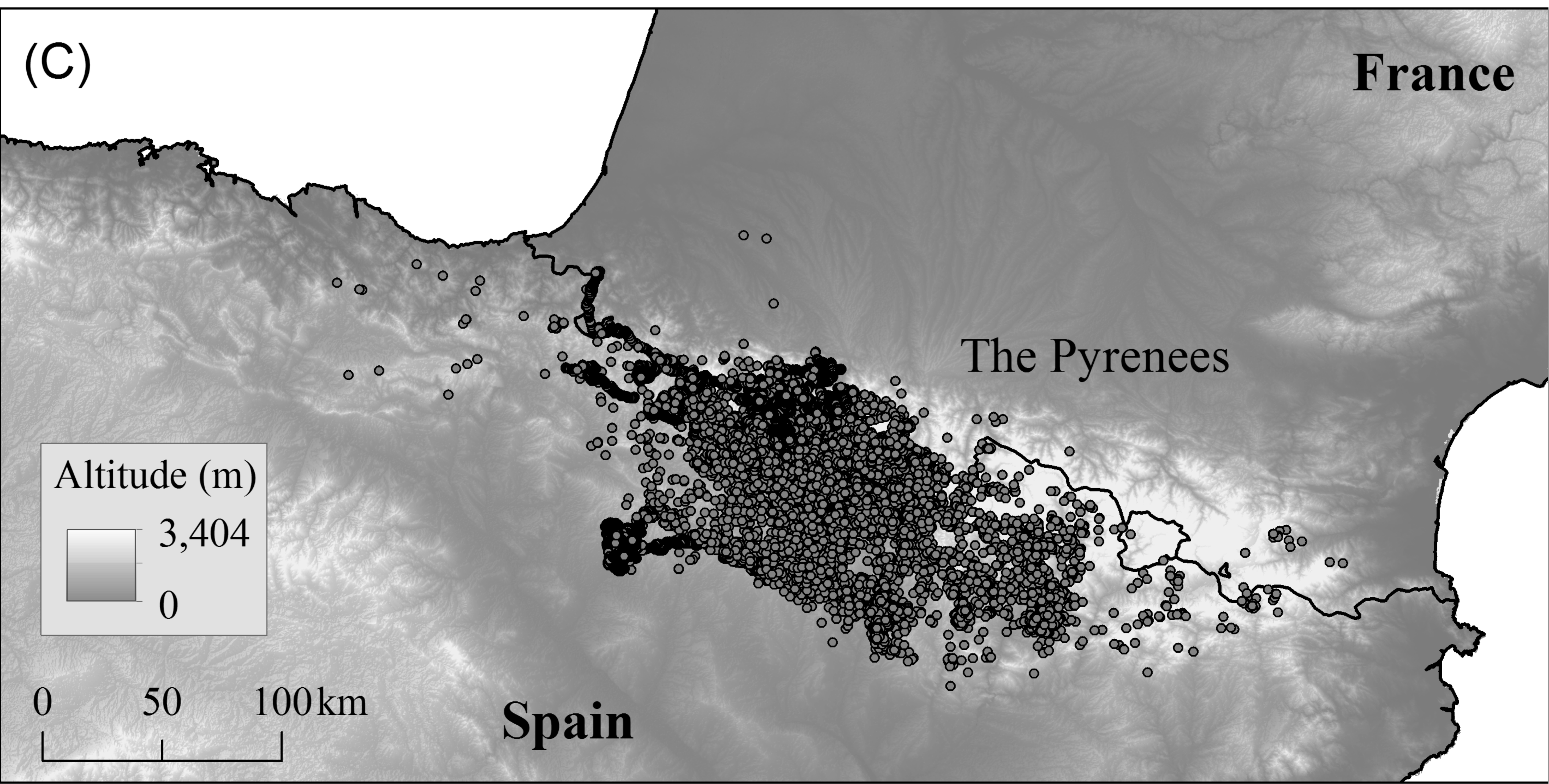

Supplement: S1 Fig — Cantabrian Mountains (B) and The Pyrenees (C). The GPS fixes (83,231) used in this study are shown as small dots. Two bearded vultures were tracked in the Cantabrian Mountain (B) using CTT tags and 11 were tracked in the Pyrenees (C) (three CTTs and eight PTTs). (PDF) [file pone.0185344.s001.pdf]

● CTT ○ OPTT

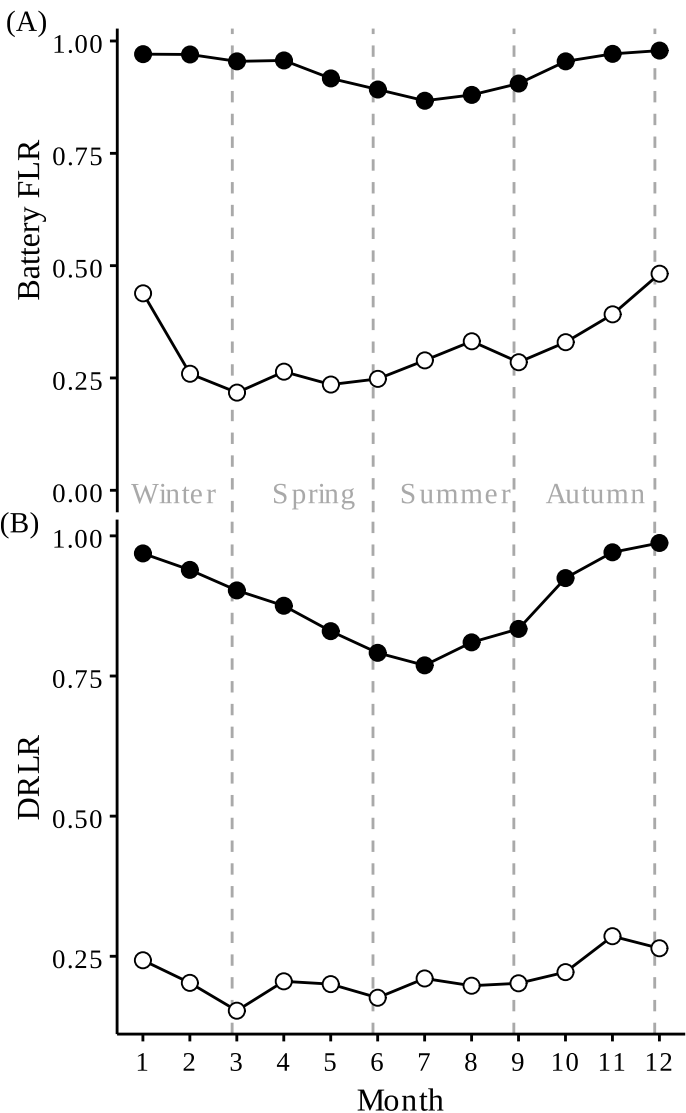

Supplement: S2 Fig — (A) Mean monthly fix-loss rate due to battery drain (Battery FLR). (B) Mean monthly data retrieval-loss rate (DRLR). In both, (A) and (B) Celltracktech GSM-GPRS tags (CTTs, n = 5) are represented as black dots and Microwave Argos tags (PTTs, n = 8) are represented as white dots. (PDF) [file pone.0185344.s002.pdf]

●CTT ○OPTT

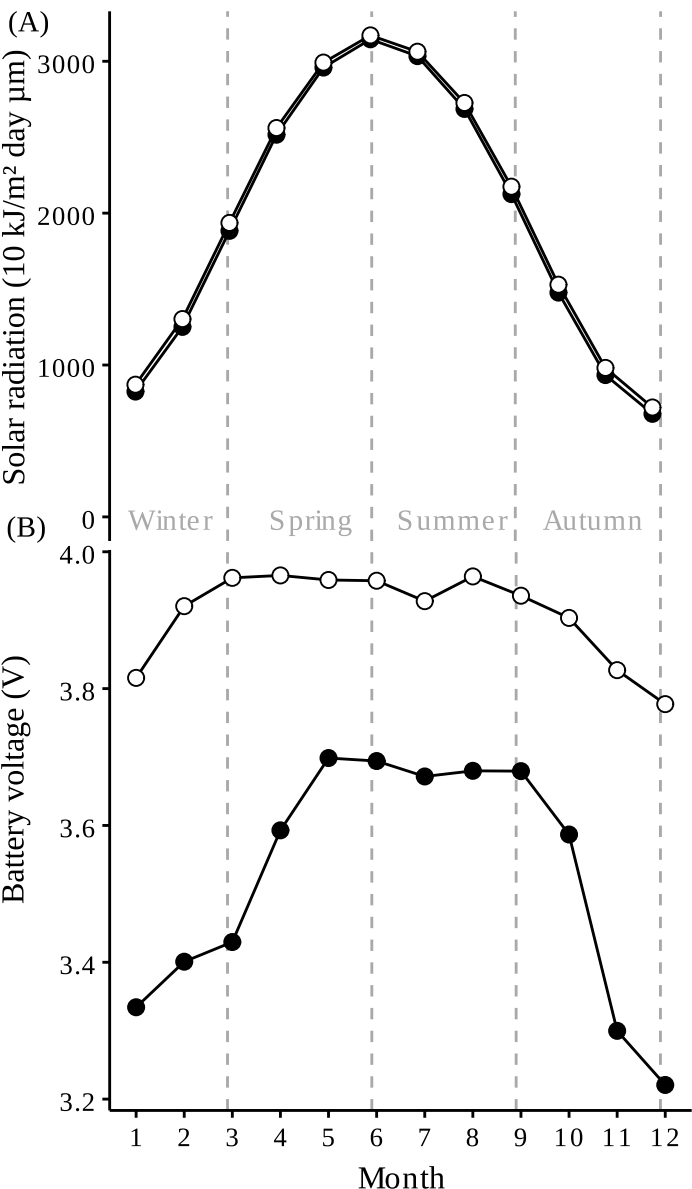

Supplement: S3 Fig — (A) Mean Monthly Potential Solar Radiation (Solar radiation). (B) Mean Monthly Battery Voltage (Battery voltage). In both, (A) and (B) Celltracktech GSM-GPRS tags (CTTs, n = 5) are represented as black dots and Microwave Argos tags (PTTs, n = 8) are represented as white dots. (PDF) [file pone.0185344.s003.pdf]

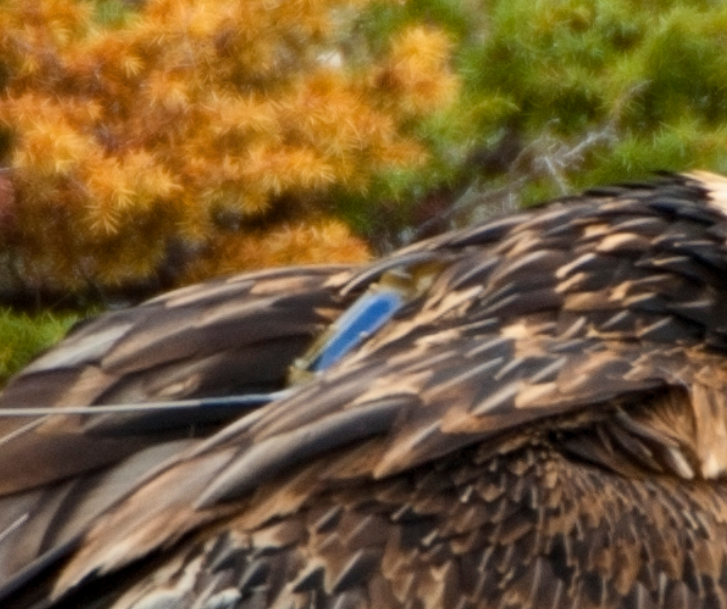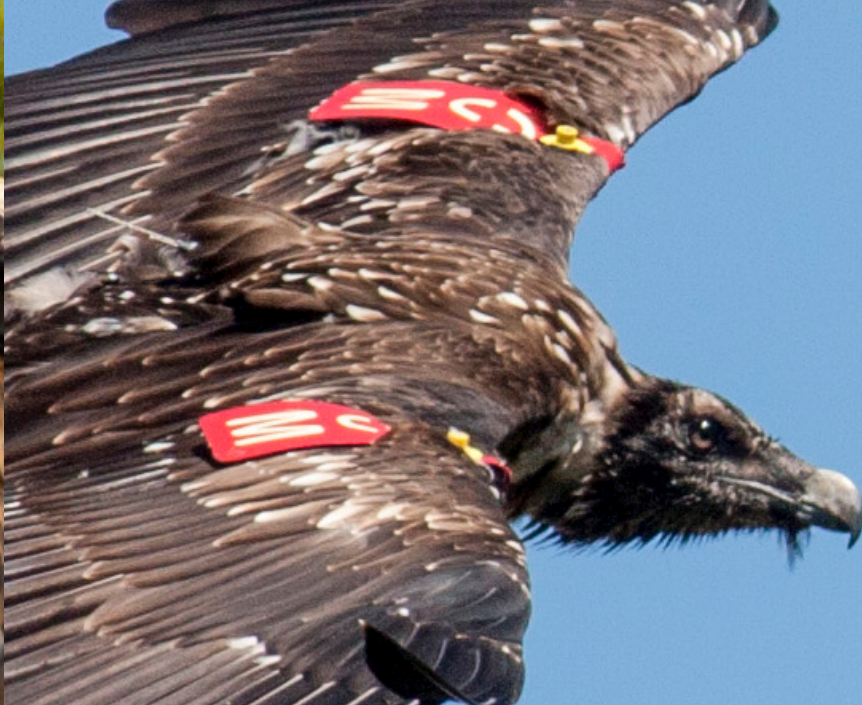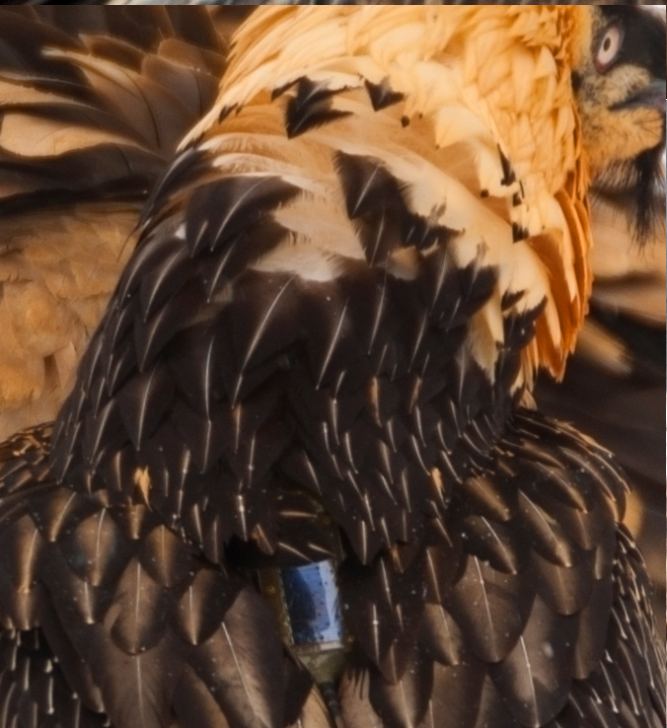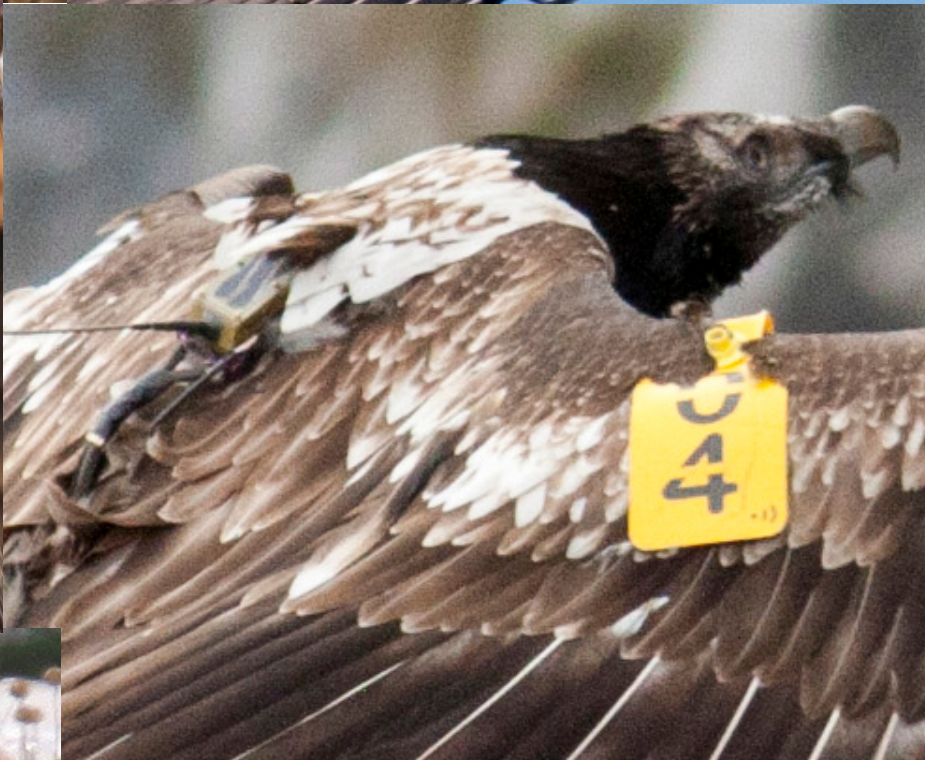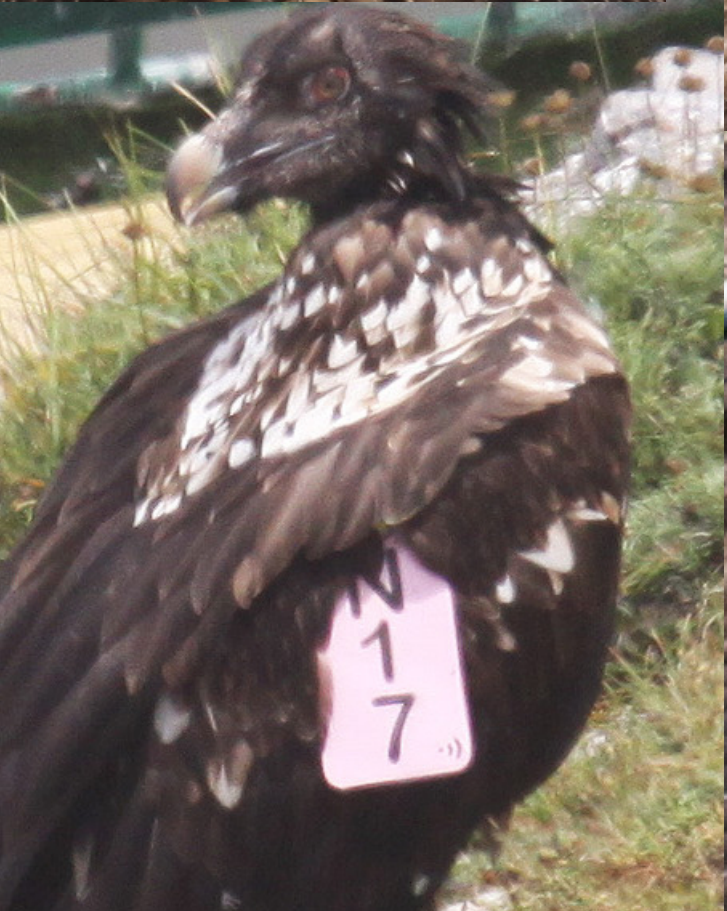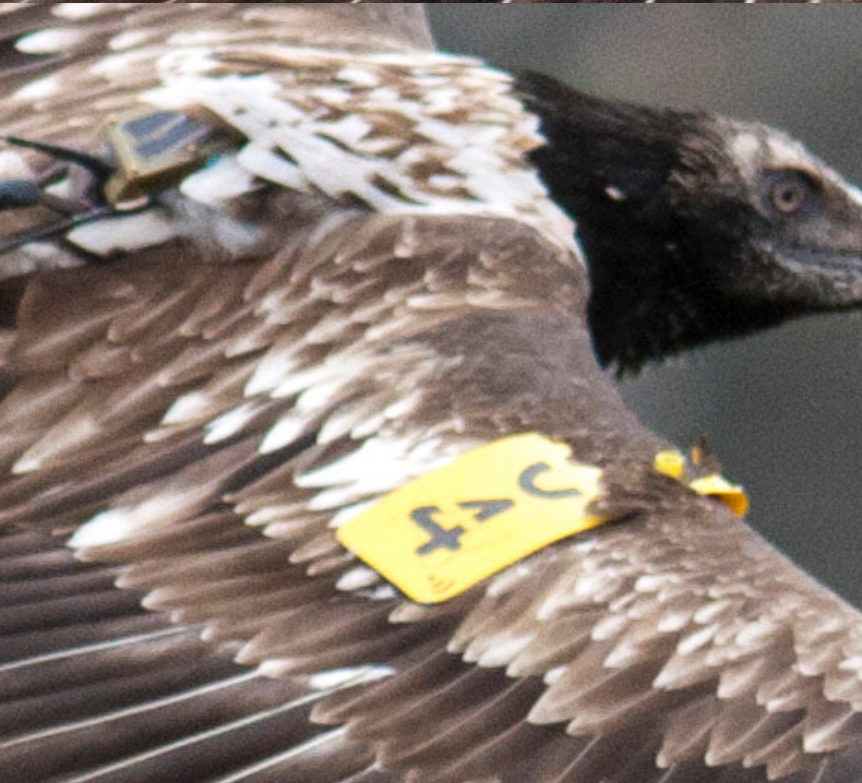

Supplement: S4 Fig — On the left individuals perching, on the right individuals flying. Frequently feathers are observed totally or partially covering the solar panels or even the tracking device completely. (C) Javier Gil Vaquero/ F.C.Q. (PDF) [file pone.0185344.s004.pdf]
