# Supplementary material for: Seasonal and circadian biases in bird tracking with solar GPS-tags
Source: PLoS One. 2017 Oct 11;12(10):e0185344. doi: 10.1371/journal.pone.0185344 (PMC5636103; doi:10.1371/journal.pone.0185344)
Supplement: S1 Table — Age code at deployment date: nestling (1), immature (3) or adult (4). Sex as F (Female) or M (Male). (PDF) [file pone.0185344.s005.pdf]

Table S1.

Individual	Data Retrieval system	Tag model	Duty cycle	Tag status	Age	Sex	Geographic region	Rearing method
Carrodilla	ARGOS	PTT-100	PTT#1	Off	4	F	Pyrenees	Wild
Asterix	ARGOS	PTT-100	PTT#1	On	1	M	Pyrenees	Hacking
Goriz	ARGOS	PTT-100	PTT#1	On	1	M	Pyrenees	Wild
Rover	ARGOS	PTT-100	PTT#1	Off	4	M	Pyrenees	Wild
Eva	ARGOS	PTT-100	PTT#1	On	4	F	Pyrenees	Wild
Ixeia	ARGOS	PTT-100	PTT#1	Off	3	F	Pyrenees	Wild
Sevil	ARGOS	PTT-100	PTT#1	On	1	F	Pyrenees	Wild
Maria	ARGOS	PTT-100	PTT#1	On	1	F	Pyrenees	Hacking
Deva	GSM/GPRS	CTT-1100	CTT#1	On	1	F	Cantabrian Mountain	Hacking
Coto	GSM/GPRS	CTT-1100	CTT#1	On	1	M	Pyrenees	Wild
Luisa	GSM/GPRS	CTT-1100	CTT#2	On	1	F	Pyrenees	Hacking
Cotiella	GSM/GPRS	CTT-1100	CTT#3	On	1	F	Pyrenees	Wild
Atilano	GSM/GPRS	CTT-1100	CTT#3	On	1	M	Cantabrian Mountain	Hacking
