# Supplementary material for: Seasonal and circadian biases in bird tracking with solar GPS-tags
Source: PLoS One. 2017 Oct 11;12(10):e0185344. doi: 10.1371/journal.pone.0185344 (PMC5636103; doi:10.1371/journal.pone.0185344)
Supplement: S2 Table — Number of GPS fixes recorded refers to the total amount of GPS data acquired by each individual for the entire tracking period. (PDF) [file pone.0185344.s006.pdf]

Table S2.

		Tracking period Dates & Timestamps (UTC)								
Individual	Duty cycle	Deployment	Start	First data retrieval	First GPS fix	Last data retrieval	Last GPS fix	End	Number of tracking months	Number of GPS fixes recorded
Carrodilla	PTT#1	2006-12-20	2007-01-01	2008-04-26	2007-01-06 14:00	2012-11-15	2012-10-28 14:00	2012-11-15	71	5125
Asterix	PTT#1	2009-06-01	2009-09-01	2009-09-03	2009-09-01 16:00	2014-06-29	2014-06-30 18:00	2014-06-30	59	7595
Goriz	PTT#1	2009-06-03	2009-09-01	2009-09-03	2009-09-01 7:00	2014-06-29	2014-06-30 19:00	2014-06-30	59	7489
Rover	PTT#1	2009-09-22	2009-10-01	2009-10-05	2009-10-01 7:00	2010-07-02	2010-06-29 19:00	2010-07-02	9	1292
Eva	PTT#1	2009-10-21	2009-11-01	2009-11-02	2009-11-01 7:00	2014-06-27	2014-06-30 19:00	2014-06-30	57	6585
Ixeia	PTT#1	2009-10-28	2009-11-01	2009-11-03	2009-11-01 7:00	2012-03-31	2012-03-29 15:00	2012-03-31	29	3480
Sevil	PTT#1	2011-06-13	2011-09-01	2011-09-02	2011-09-01 7:00	2014-06-30	2014-06-30 19:00	2014-06-30	34	5054
Maria	PTT#1	2011-07-07	2011-09-01	2011-09-02	2011-09-01 7:00	2014-06-30	2014-06-30 15:00	2014-06-30	34	5335
Deva	CTT#1	2010-06-26	2010-09-01	2011-04-05	2011-04-05 11:46	2014-06-30	2014-06-30 12:03	2014-06-30	39	17699
Coto	CTT#1	2010-06-30	2010-09-01	2011-01-18	2011-01-18 11:26	2014-06-08	2014-06-08 13:12	2014-06-30	41	10765
Luisa	CTT#2	2011-07-07	2011-09-01	2011-09-20	2011-09-01 9:49	2014-06-23	2014-06-30 16:07	2014-06-30	34	5784
Cotiella	CTT#3	2012-06-15	2012-09-01	2012-09-03	2012-09-03 8:29	2014-01-22	2014-01-22 12:05	2014-06-30	17	5684
Atilano	CTT#3	2012-07-02	2012-09-01	2012-09-04	2012-09-01 10:58	2014-06-16	2014-06-16 13:10	2014-06-30	22	1344
