# Supplementary material for: Seasonal and circadian biases in bird tracking with solar GPS-tags
Source: PLoS One. 2017 Oct 11;12(10):e0185344. doi: 10.1371/journal.pone.0185344 (PMC5636103; doi:10.1371/journal.pone.0185344)
Supplement: S3 Table — (PDF) [file pone.0185344.s007.pdf]

Table S3.

Individual	Data Retrieval system	Duty cycle	Data retrieval time lag (days)					Fix time lag					
			max	mean	SD	median	mode	min (seconds)	max (days)	mean (hours)	SD (hours)	Median (minutes)	Mode (minutes)
Carrodilla	ARGOS	PTT#1	22	3	2	2	2	3600	9	4.74	13.86	120	120
Asterix	ARGOS	PTT#1	14	2	1	2	2	3600	8	3.15	5.35	120	120
Goriz	ARGOS	PTT#1	14	2	1	2	2	3600	9	4.28	6.95	120	120
Rover	ARGOS	PTT#1	10	2	1	2	2	3600	8	4.14	7.59	120	120
Eva	ARGOS	PTT#1	12	3	1	2	2	3600	11	4.82	8.31	120	120
Ixeia	ARGOS	PTT#1	14	2	1	2	2	3600	11	4.52	8.36	120	120
Sevil	ARGOS	PTT#1	10	2	1	2	2	7200	7	4	5.59	120	120
Maria	ARGOS	PTT#1	10	2	1	2	2	3600	7	3.8	5.41	120	120
PTT mean			13	2	1	2	2	4050	9	4	8	120	120
PTT SD			4	0	0	0	0	1273	2	1	3	0	0
Deva	GSM/GPRS	CTT#1	246	7	25	1	1	1	243	1.82	65.79	0.63	0.63
Coto	GSM/GPRS	CTT#1	230	14	40	1	1	1	230	2.72	85.19	0.63	0.57
Luisa	GSM/GPRS	CTT#2	59	14	8	12	10	55	12	3.67	8.49	29.45	15.63
Cotiella	GSM/GPRS	CTT#3	183	4	18	1	1	54	182	1.89	62.15	15.42	15.42
Atilano	GSM/GPRS	CTT#3	310	13	49	2	1	54	307	8.27	196.62	16.31	15.03
CTT mean			206	11	28	3	3	33	195	4	84	12	9
CTT SD			94	5	16	5	4	29	111	3	69	12	8
